# IgG4-Seronegative Autoimmune Pancreatitis and Sclerosing Cholangitis

**DOI:** 10.1155/2015/591360

**Published:** 2015-08-25

**Authors:** Allon Kahn, Anitha D. Yadav, M. Edwyn Harrison

**Affiliations:** ^1^Department of Medicine, Mayo Clinic, Scottsdale, AZ, USA; ^2^Division of Gastroenterology and Hepatology, Mayo Clinic, Scottsdale, AZ, USA

## Abstract

IgG4-related disease is a relatively novel clinical entity whose gastrointestinal manifestations include type 1 autoimmune pancreatitis (AIP) and IgG4-associated sclerosing cholangitis. The presence of elevated serum IgG4 is suggestive but not essential for the diagnosis of type 1 AIP and is a pervasive feature of the proposed diagnostic criteria. The differential diagnosis of type 1 AIP includes malignant conditions, emphasizing the importance of a deliberate, comprehensive evaluation. Management of patients with a suggestive clinical presentation, but without serum IgG4 elevation, is difficult. Here we present three cases of IgG4-seronegative AIP and sclerosing cholangitis that responded to empiric steroid therapy and discuss approach considerations. These cases demonstrate the value of meticulous application of existing diagnostic algorithms to achieve a clinical diagnosis and avoid surgical intervention.

## 1. Introduction

Type 1 autoimmune pancreatitis (AIP) and immunoglobulin G4- (IgG4-) associated sclerosing cholangitis (IgG4-SC) are pancreatic and biliary manifestations of IgG4-related disease, respectively. Recently, IgG4-related disease has emerged as a novel clinical entity characterized by multiorgan infiltration of IgG4-positive cells, storiform fibrosis, and elevated serum IgG4 levels. Several diagnostic criteria have been proposed in the last decade based on radiology, histopathology, serology, and response to steroids [1–3]. However, the diagnosis still remains challenging. Often the differential diagnosis for AIP/IgG4-SC includes malignant conditions such as pancreatic cancer and cholangiocarcinoma, placing patients at risk for unnecessary surgery for a more benign condition.

Current clinical diagnostic criteria for type 1 AIP/IgG4-SC involve elevated serum IgG4 concentrations (>135 mg/dL). The reported sensitivity of increased serum IgG4 levels in AIP ranges from 68% to 81% [1, 4]. Hence, in patients with normal serum IgG4 levels, a thorough workup including pancreatic and biliary imaging, histology, and identification of extrapancreatic manifestations aids in accurate diagnosis. Here we describe three cases of serum IgG4 negative type 1 AIP/IgG4 SC that responded to a trial of steroid therapy, allowing for the avoidance of surgery.

## 2. Case 1

An 80-year-old Caucasian man with a history of resected prostate adenocarcinoma and hyperlipidemia presented with complaints of pruritus, anorexia, fatigue, 20 pound weight loss, and painless jaundice. He denied abdominal pain, melena, or hematochezia. His vital signs were within normal limits and physical examination revealed only jaundice. There was no history of significant alcohol use.

**Figure 1:**
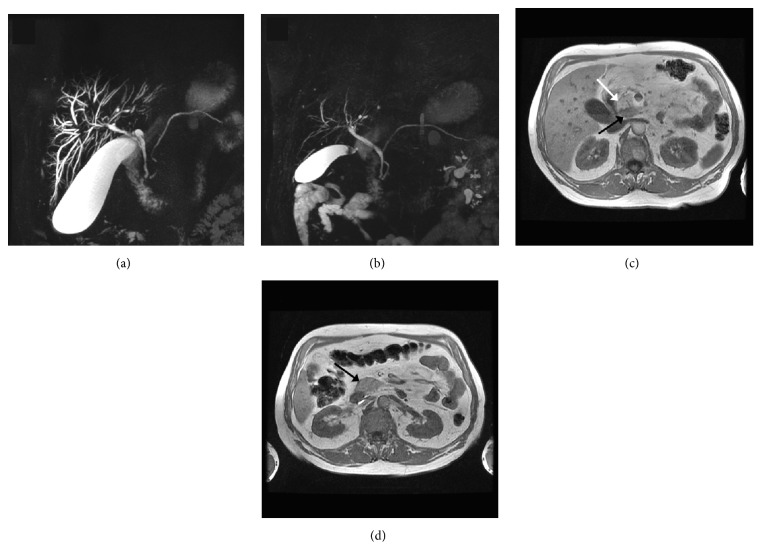
MRCP findings: Case 1. (a) Narrowing and stricture of the common bile duct with dilated intrahepatic ducts. (b) Resolution of intrahepatic duct dilation and CBD narrowing after steroid therapy. (c) 2.7 cm hypointense lesion in the pancreatic head (black arrow) near a region of normal pancreatic parenchyma (white arrow). (d) Resolution after steroid therapy. The pancreatic parenchyma is homogeneous and isointense with the adjacent liver.

Laboratory workup revealed an elevated total bilirubin 8.5 mg/dL, direct bilirubin 5.2 mg/dL, alkaline phosphatase 372 IU/L, alanine aminotransferase (ALT) 299 IU/L, aspartate aminotransferase (AST) 143 IU/L, amylase 70 *μ*/L, and lipase 54 *μ*/L. His platelet count was 258 and International Normalized Ratio (INR) 0.9. Carbohydrate antigen (CA) 19-9 was 175 U/mL and carcinoembryonic antigen (CEA) level 1.1 ng/mL. Total IgG was 1,550 mg/dL and IgG4 levels were 51.8 (reference range 2.4–121). Other serologic tests included anti-neutrophil antibody (ANA), anti-smooth muscle antibody (ASMA), and anti-mitochondrial antibody (AMA), all of which were negative. A computed tomography (CT) of the abdomen demonstrated diffuse intrahepatic biliary ductal dilatation. Magnetic resonance imaging (MRI) revealed an area of enhancement concerning for neoplasm at the biliary confluence, narrowing of the central right and left intrahepatic bile ducts and intrahepatic biliary duct dilation (Figure 1(a)). Diffuse pancreatic enhancement was noted. An ERCP was performed and demonstrated a complex narrow biliary stricture involving both right and left hepatic ducts and extending to the common hepatic duct, consistent with type 4 pattern cholangiography [5]. Biliary brushings and FISH were negative for malignancy. IgG4 immunostaining of biliary brushing specimen was not pursued. Subsequently our patient underwent three ERCPs with biliary dilation/stent exchange and biliary brushings for cytology and FISH, all of which revealed no evidence of malignancy.

Six months after initial presentation, a repeat MRI was obtained which demonstrated a new 2.7 cm mass at the posterior aspect of the head of the pancreas extending into the uncinate process (Figure 1(c)). An endoscopic ultrasound with fine needle aspiration was performed and revealed benign pancreatic ductal cells and acini in addition to fragments of chronically inflamed stromal material. IgG4 immunostaining showed rare IgG4-positive inflammatory cells and was felt to be inconclusive. Periampullary mucosal biopsies showed small bowel mucosa without diagnostic abnormality. Incidentally, a CT of the chest revealed bilateral hilar, paratracheal and mediastinal enlarged lymph nodes, small pulmonary nodules, and ground glass opacities.

After a thorough negative workup for malignancy, based on the available clinical, radiological, and extrapancreatic presentation, a diagnosis of type 1 AIP and IgG4-SC was suspected. The patient was started on 40 mg of prednisone and continued for 4 weeks and gradually tapered. His symptoms abated and a follow-up MRI performed demonstrated complete resolution of the pancreatic mass and biliary strictures (Figures 1(b) and 1(d)). With this clinical and radiologic response, the patient was diagnosed with IgG4-related SC and the symptoms have not recurred after 3 years of follow-up.

## 3. Case 2

**Figure 2:**
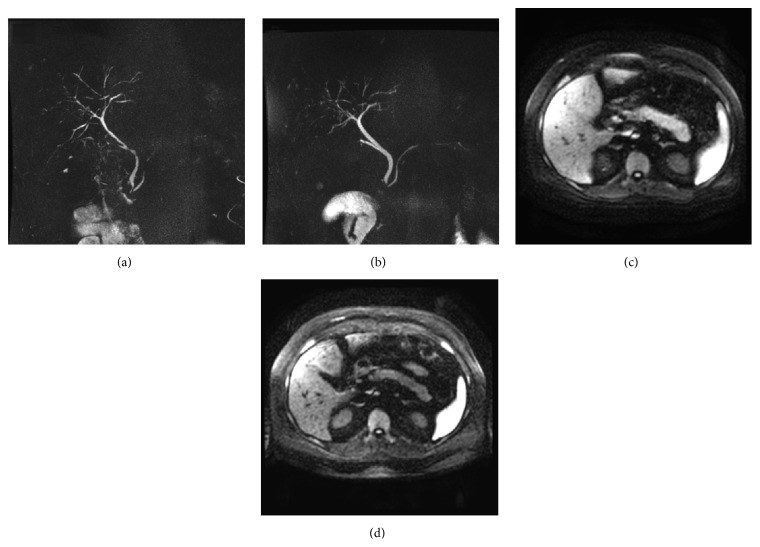
MRCP findings: Case 2. (a) Extrahepatic bile duct narrowing. (b) Resolution after steroid therapy. (c) Diffusion restriction shows hyperintensity and edema in the pancreatic tail. (d) Resolution after steroid therapy.

A 45-year-old gentleman with prior history of small duct primary sclerosing cholangitis and Crohn's disease presented with symptoms of abdominal pain, pruritus, dark urine, and jaundice. Laboratory analysis showed an AST of 152, ALT of 158, alkaline phosphatase of 531, total bilirubin of 5.6, direct bilirubin of 3.8, total protein of 7.4, albumin of 4.0, lipase of 157, amylase of 82, CA of 19-9 194, CEA of 2.9, total IgG of 1620, and IgG4 of 53.3. MRI of the abdomen demonstrated diffuse enlargement of the body and the tail of the pancreas. No focal enhancing pancreatic mass was identified. MRCP revealed new irregular narrowing of the common hepatic duct, upper common bile duct beaded dilatation, and narrowing of the intrahepatic bile ducts (Figures 2(a) and 2(c)). Several prominent porta hepatis lymph nodes were noted.

An ERCP was performed and revealed mild intrahepatic biliary strictures and moderately severe common bile duct stricture, most consistent with a type 2 cholangiogram [5]. Sphincterotomy and dilation were performed and brushings were obtained. Biliary brushings revealed reactive epithelial cells and FISH was negative for malignancy; IgG4 immunostaining was not performed. Based on the clinical findings and parenchymal and ductal imaging, a diagnosis of autoimmune pancreatitis and IgG4-SC was made and patient was initiated on prednisone 60 mg daily. Within 2 weeks of initiating steroid therapy, his laboratory tests show significant improvement with resolution of cholestasis and MRCP showed significant improvement in intrahepatic and extrahepatic bile duct changes with resolution of pancreatic enlargement (Figures 2(b) and 2(d)). A decision was made to continue immunosuppressive therapy and he was started on mycophenolate 500 mg twice daily (prior history of Imuran-induced pancreatitis). His steroids were tapered gradually and stopped with no subsequent relapse.

## 4. Case 3

A 52-year-old gentleman presented to the gastroenterology clinic with a history of acute pancreatitis and complaints of abdominal pain. He denied abnormal weight loss or alcohol use. Laboratory analysis showed an amylase of 495, lipase of 1305, bilirubin of 2.8, AST of 658, ALT of 533, and alkaline phosphatase of 65. He was diagnosed with acute pancreatitis and treated conservatively. Abdominal MR imaging revealed segmental biliary dilatation from the bifurcation proximally to the right and left system with CBD stricture. The common hepatic duct was 10 mm and the CBD distally was 2.5 mm. The pancreatic duct was unremarkable. Additionally, there was significant retroperitoneal adenopathy and diffuse pulmonary nodules on the imaging.

An ERCP was performed and revealed a 2.5 cm high-grade stricture in the distal common bile duct, consistent with type 1 cholangiogram [5]. Brushing and biopsies were taken and a biliary stent was placed. A CA 19-9 at the time was 37. The biliary biopsies and FISH were negative for malignancy. IgG4 immunostaining of biliary brushings demonstrated occasional, scattered positive cells of indeterminate clinical significance. An EUS performed revealed a hypoechoic mass in the head of the pancreas (measuring 26 × 18 mm) and the body (measuring 22 × 22 mm). Pancreatic biopsies showed mild atypical epithelial cells. Serum total IgG was elevated at 2,430, but IgG4 level was normal at 41.2. On follow-up, he was noted to have enlarged submandibular salivary glands. Given a negative diagnostic workup, a CT guided biopsy of submandibular glands was pursued which confirmed chronic inflammatory infiltrate with numerous IgG4 positive plasma cells. A diagnosis of type 1 AIP and IgG4-SC was made and the patient was treated with prednisone 40 mg daily. He was also started on Imuran 150 mg daily and his prednisone was slowly tapered and stopped. The patient improved clinically, his laboratory parameters normalized, and repeat imaging showed dramatic improvement in biliary strictures with a normal-appearing pancreas.

## 5. Discussion

IgG4-related disease is a multiorgan fibroinflammatory disorder characterized by organ infiltration with lymphoplasmacytic cells rich in IgG4, storiform fibrosis, obliterative phlebitis, elevated serum IgG4 levels, and response to steroids. Two types of AIP have been described in the literature. Type 1 AIP is related to IgG4-related disease, while type 2 (idiopathic duct centric pancreatitis) is associated with a distinct characteristic histological pattern and is usually not accompanied by extrapancreatic manifestations [6]. Type 1 AIP is prevalent worldwide and typically affects middle-aged and elderly men. The most common presentation of type 1 AIP is obstructive jaundice. Other symptoms can include abdominal pain, weight loss, and anorexia [7]. Additionally, AIP can present as acute and chronic pancreatitis and could closely mimic pancreatic cancer. The commonest extrapancreatic manifestation of type 1 AIP is IgG4-SC, which can mimic cholangiocarcinoma. Other extrapancreatic manifestations include salivary gland enlargement, lymphadenopathy, pulmonary disease with nodules, hydronephrosis, and retroorbital disease [8].

In our current series, we describe three patients with type 1 AIP and IgG4-SC with normal serum IgG4 levels in whom steroid treatment was successful and surgery was entirely avoided. Each of these cases provides lessons in the diagnosis of IgG4-SC in patients with normal IgG4 levels. In Case 1, cholangiocarcinoma was suspected on the basis of clinical findings and compatible radiologic findings on CT, MR, and ERCP. However, a thorough and measured approach to diagnosis led to four ERCPs with biliary brushings and FISH, which were negative for malignancy. This allowed the patient to defer needless surgery. Follow-up MRI after six months revealed a new pancreatic mass which would not be expected with cholangiocarcinoma, and pulmonary nodules and mediastinal lymphadenopathy were seen on CT imaging, suggesting extrapancreatic involvement. When an EUS with FNA of the pancreatic mass did not reveal malignancy, but rather a chronic stromal infiltrate, the combination of pathologic findings and extrapancreatic involvement led to a trial of prednisone. With dramatic clinical and radiologic response to steroids, the diagnosis of type 1 AIP was made and surgery was avoided.

In Case 2, the patient presented with abdominal pain and jaundice. Imaging showed diffuse enlargement of the pancreas, as well as biliary strictures and pulmonary nodules. Serological tests were negative for IgG4, and histology and FISH were negative from ERCP brushings. Based on clinical and radiological presentation, a diagnosis of AIP was considered and empiric treatment was initiated with steroids. The patient responded well, with resolution of pancreatic enlargement and biliary strictures on imaging.

In our 3rd case, the patient presented with a diagnosis of acute pancreatitis of unknown etiology. His abdominal imaging, however, revealed biliary strictures which raised the question of an uncommon cause of pancreatitis. By carefully pursuing this clinical clue, misdiagnosis was avoided. Two ERCPS with biliary brushings and FISH were negative for malignancy, and EUS demonstrated pancreatic masses in the head and the body of the pancreas, biopsies of which were also negative for malignancy. The subsequent detection of an enlarged submandibular gland allowed a diagnostic biopsy that demonstrated chronic inflammatory infiltrate with numerous IgG4 positive plasma cells. This patient also was treated successfully with steroids, and continues on Imuran with no relapse.

The diagnosis of AIP is challenging and a number of diagnostic criteria (Japan, Italy, the United States, and Korea) have been proposed in the recent years. The HISORt criteria proposed by the Mayo Clinic to diagnose AIP include histology, imaging findings, elevated serum IgG4 levels, other organs involvement, and response to steroids [1]. In order to unify the disparate diagnostic criteria, a multinational group convened in 2011 and developed International Consensus Diagnostic Criteria (ICDC) for AIP [3]. The ICDC criteria are based on the following parameters: pancreatic parenchymal imaging, pancreatic ductal imaging (ERCP), serum IgG4 level, other organs involvement, histology of the pancreas, and response to steroid treatment. These parameters may provide level 1 (highly suggestive) or level 2 (supportive) evidence that will aid in definitive diagnosis and are associated with well-defined diagnostic algorithms.

The ICDC criteria can still be successfully applied in cases of seronegative IgG4-associated AIP and IgG4-SC. In Case 1, MRI pancreatography findings were indeterminate for AIP. A thorough evaluation for possible malignancy was negative. Pancreatic ductal imaging was normal. ERCP revealed level 1 findings, but serology was negative and no additional organ involvement (OOI) was noted. Two level 1 criteria were not met, so EUS-guided pancreatic biopsy was performed and was inconclusive. Because of the presence of a single level 1 OOI, steroid trial was pursued and resulted in radiographic and symptomatic resolution, supporting a diagnosis of type 1 AIP. In Case 2, initial MR pancreatography was typical for type 1 AIP. There was a single level 1 OOI criterion (multiple proximal and distal CBD strictures); thus, a steroid trial was pursued and resulted in substantial improvement, confirming the diagnosis. In Case 3, neither pancreatic parenchymal imaging nor ductal imaging was typical for AIP. However, level 1 OOI findings were seen on ERCP. A pancreatic biopsy was thus performed and yielded inconclusive findings. At this point, a steroid trial would have been reasonable; however, the interval development of submandibular gland enlargement allowed for establishment of definitive histological diagnosis.

The typical histological findings in type 1 AIP are comprised of abundant infiltration of lymphocytes, IgG4 positive plasma cells (greater than 10 IgG4 positive cells per high power field), fibrosis in periductal and interlobular areas, and obliterative phlebitis [9]. Tissue samples obtained with EUS-guided pancreatic trucut biopsies provide accurate histological diagnosis as compared to FNA alone [10]. However, if tissue samples are not available, the diagnosis can be made with the aid of imaging studies, extrapancreatic involvement, and response to steroids [2, 11]. In our series, two patients did not have definitive histopathological findings of lymphoplasmacytic infiltrate with IgG4 cells. Yet, based on the collateral evidence, all patients were treated with steroids and immunosuppressive medications and thus surgery was avoided.

Increased serum IgG4 levels (>135 mg/dL) have been frequently described in patients with type 1 AIP and have been included as a part of diagnostic criteria. Hamano and colleagues first described an association between elevated IgG4 and AIP in 2001 and reported the sensitivity and specificity of elevated IgG4 for AIP as 95 and 97%, respectively [12]. However, subsequent studies of larger AIP patient cohorts demonstrated IgG4 seropositivity in as few as 76% [13]. Thus, further estimates of sensitivity and specificity for AIP diagnosis have been shown to vary widely and are reported as low as 62 and 59%, respectively [14]. This heterogeneity is largely attributable to the application of disparate diagnostic criteria in study design, a fact which prompted the ICDC's creation. Ultimately, the sole elevation of serum IgG4 is inadequate to establish a definitive diagnosis of AIP. Some have suggested that it may play a clearer role in the differentiation of AIP from malignancy, particularly at higher cutoff levels where specificity is enhanced [14].

It has also been suggested that IgG4-seronegative AIP may behave as a different clinicopathologic entity. When compared to IgG4-seropositive patients, those with negative serology are more likely to be female, present with abdominal pain or acute pancreatitis, and demonstrate segmental pancreatic body and/or tail enlargement [15, 16]. They are less likely to present with obstructive jaundice and less frequently require maintenance immunosuppression, owing to a lower relapse rate [17]. In this way, IgG4 level may serve as a prognostic biomarker. Patients with IgG4 seropositivity are also noted to have a higher prevalence of extrapancreatic manifestations of IgG4-related disease, particularly lacrymal and salivary gland involvement and hilar lymphadenopathy [17]. In contrast to this data, all of our IgG4-seronegative patients were male and presented with extrapancreatic disease (i.e., IgG4-SC and submandibular involvement), with 2 of 3 presenting with obstructive jaundice and requiring maintenance immunosuppression. This divergence from previously documented patterns is likely a manifestation of the observational quality of existing data and the heterogeneous clinical course of this relatively novel disease.

In Cases 1 and 3, pancreatic imaging was not suggestive of typical features for AIP, while cholangiography suggested the presence of IgG4-SC. While AIP and IgG4-SC often coexist, it is important to recognize that they are likely distinct, if closely related, manifestations of the single overarching clinical entity of IgG4-related disease. Several studies have reported histologically proven IAC (i.e., lymphoplasmacytic infiltrate, storiform fibrosis, and obliterative phlebitis on resection specimen) in the absence of any clinical or radiographic evidence of AIP [18–20]. As was seen in our cases and is corroborated by the ICDC algorithms, biliary findings suspicious for IgG4-SC without characteristic evidence of AIP should not lead one away from a meticulous evaluation for IgG4-related disease.

The diagnosis of isolated IgG4-SC is challenging and IgG4 serology alone is inadequate to establish the diagnosis, as has been previously discussed. The sensitivity of IgG4 for IgG4-SC diagnosis has been estimated as 74%, a level comparable to estimates in AIP [18]. Several of the reported cases of isolated IgG4-SC demonstrate borderline elevations in IgG4 [20] and elevation of IgG4 in other stricturing biliary disorders, such as PSC, has been observed [21]. The addition of IgG4 immunostaining is often critical to accurate identification. Ampullary, hepatic, or biliary duct biopsies have been demonstrated as useful modalities, with relatively variable sensitivities (24–80%), but high specificities (91–100%) for the diagnosis of IAC [22].

Although our goal is early identification and treatment of benign IgG4-related disease and avoidance of surgery, it should be emphasized that the misdiagnosis of AIP/IgG4-SC in the setting of pancreatic cancer or cholangiocarcinoma must be strictly avoided [23]. In a recent UK study evaluating long-term outcomes in patients with AIP/IgG4-SC, 13 of 115 patients (11%) were diagnosed with a malignancy before or after the diagnosis of AIP [8]. Hence, caution should be exercised to rule out malignancy prior to embarking on investigations aimed at diagnosing AIP/IgG4-SC. On the other hand, the reported incidence of benign disease after pancreatoduodenectomy for a presumed malignancy is 5–13% and the incidence of AIP in the benign resected specimens is 30–43% [24]. Hence, diagnosis of AIP should be made cautiously taking into account all other collateral information such as characteristic imaging findings, serologic parameters, extrapancreatic involvement, and perhaps treatment with a short course of steroids prior to considering surgery. In our case series, we engaged in a thorough evaluation to exclude malignancy and include other findings within the spectrum of IgG4-related disease before diagnosis and initiation of steroid therapy.

Type 1 AIP/IgG4-SC typically responds dramatically to steroids. In our series we treated all patients with prednisone 40 mg daily for 4 weeks and tapered by 5 mg/week with close clinical follow-up, liver function tests, and imaging studies. In addition, all patients required biliary drainage procedures. Even though disease relapse is common with type 1 AIP, (30%–50%) none of our patients had clinical or biochemical relapse [25, 26]. However, in our series two out of 3 patients are maintained on immunosuppressive therapy (Imuran or mycophenolate).

In summary, our case series highlights the benefit of early recognition and medical treatment of IgG4-negative type 1 AIP and IgG4-SC. Despite normal IgG4 levels, a diagnosis of IgG4-related AIP and IgG4-SC should be considered when there is a typical clinical presentation, the presence of characteristic pancreatobiliary imaging findings, and extrapancreatic involvement. When IgG4-related AIP is recognized without elevation of IgG4, the patient can be spared morbidity associated with unnecessary surgical treatment by initiating a steroid trial and monitoring patients carefully.
